# Molecular and Cellular Mechanisms Influenced by Postbiotics

**DOI:** 10.3390/ijms222413475

**Published:** 2021-12-15

**Authors:** Rafał Jastrząb, Damian Graczyk, Pawel Siedlecki

**Affiliations:** Institute of Biochemistry and Biophysics, Polish Academy of Sciences, 02-106 Warsaw, Poland; jastrzab@ibb.waw.pl (R.J.); dgraczyk@ibb.waw.pl (D.G.)

**Keywords:** postbiotics, probiotics, mechanism of action

## Abstract

In recent years, commensal bacteria colonizing the human body have been recognized as important determinants of health and multiple pathologic conditions. Among the most extensively studied commensal bacteria are the gut microbiota, which perform a plethora of functions, including the synthesis of bioactive products, metabolism of dietary compounds, and immunomodulation, both through attenuation and immunostimulation. An imbalance in the microbiota population, i.e., dysbiosis, has been linked to many human pathologies, including various cancer types and neurodegenerative diseases. Targeting gut microbiota and microbiome–host interactions resulting from probiotics, prebiotics, and postbiotics is a growing opportunity for the effective treatment of various diseases. As more research is being conducted, the microbiome field is shifting from simple descriptive analysis of commensal compositions to more molecular, cellular, and functional studies. Insight into these mechanisms is of paramount importance for understanding and modulating the effects that microbiota, probiotics, and their derivatives exert on host health.

## 1. Introduction

When dealing with the human microbiome and its influence on human health, we are considering about 100 times more bacterial genes than present in the human genome [1]. It is not only the products of these genes that can interact with the host. Components of bacterial cells, such as lipopolysaccharides (LPS) [2], or metabolites, such as short-chain fatty acids (SCFA), efficiently target host metabolic and immune systems [3,4]. The host is also capable of influencing the composition of the microbiome, e.g., by regulating the synthesis of specific proteins [5] or by expressing specific receptor variations [6]. Such cross-talk interactions reveal the dynamic states contributing to homeostasis between the bacterial community and the host. An increasing amount of research indicates that it is possible to modulate this state by the administration of probiotics or bacterial byproducts.

Several strategies have been suggested to regulate gut microbiome dysbiosis; among them are antibiotics, prebiotics, and probiotics [7]. Probiotics are live microorganisms that confer health benefits on the host [8]. Their impact on host health is now commonly recognized, with evidence of immunomodulatory [9,10], hypocholesterolemic [11], anti-obesogenic [12], anti-hypertensive [13], anti-proliferative [14], anti-neurodegenerative [15,16], and antioxidant effects [17]. Probiotic administration has its limitations, mainly due to the potential risk of infection/bacteremia [18] and the possibility of transmitting antibiotic resistance genes (extensively discussed in [19,20]). Non-viable probiotics, called postbiotics, may present a safer alternative with similar physiological effects [21,22]. The term ”postbiotics” is relatively new and refers to non-living microorganisms and their cell components, with or without metabolism products; in this sense, postbiotics distinguishes itself from the pro and synbiotics definitions [23]. In the literature, a few synonymous terms can be found, such as paraprobiotics, ghost probiotics, non-viable probiotics, or inactivated/non-viable microbial cells [24,25,26]. Postbiotic administration is a novel, promising strategy for a growing number of diseases, especially for those that affect the immune system [27,28]. Here, we review the influence of postbiotic elements (Table 1) on cellular signaling pathways, summarize the strategies of postbiotic usage, and discuss their current pitfalls. We show that from a technological point of view, postbiotics may be an interesting source of raw materials/active pharmaceutical ingredients (APIs) for drug discovery and production processes. We specifically focus on interactions of specific postbiotic elements (and their combination) with molecular targets, which we believe is the route to a safer and more controllable alternative to live probiotic administration.

This review provides granular information about the origin and routes of interaction of postbiotics with host receptors. Emphasis is placed on the relationship between structure and activity, and how it can explain some of the contradictory results obtained by the field. We also discuss the interplay between cellular pathways, which is crucial for shaping the final immunological response to postbiotics. We show the impact of experimental design on the obtained results and finally, we hypothesise how to broaden the application of postbiotics-derived factors for use in human health care.

## 2. The Impact of Postbiotics on Immunomodulatory Pathways

One of the best-described pro-health properties of postbiotics is their immunomodulatory potential. In a recent study performed on patients with atopic dermatitis, postbiotic administration (of heat-killed *Lactobacillus rhamnosus* IDCC 3201) led to the remission of symptoms and reduced overall systemic inflammation [60]. In such studies, however, the observed effects are a result of multi-ingredient postbiotic fractions in which the compounds are not strictly defined. A potential immunomodulatory effect could originate from a peptide/protein, cell wall components such as lipoteichoic acid (LTA), small molecules (i.e., short-chain fatty acids (SCFA)), or a combination of these elements [61,62]. These studies often do not clarify which fraction is responsible for the observed health properties. This frequently leads to complex cellular responses, which often are contradictory depending on the origin and composition of the tested material, as well as the experimental settings used. Below, we summarize the impact of postbiotics on the typical pathways involved in immune responses, and where possible, we describe the molecular mechanisms.

### 2.1. NF-κB and MAPK Pathways

Inflammation is one of the reactions of organisms to infection or tissue injury. However, prolonged inflammation is a source of harmful effects observed in many inflammatory diseases, such as inflammatory bowel disease (IBD), arthritis, gastritis, asthma, or atherosclerosis. The common characteristic feature of these diseases is dysregulation of the nuclear factor kappa-light-chain-enhancer of activated B cell (NF-κB) and/or mitogen-activated kinase (MAPK) pathways [63,64,65,66].

The NF-κB complex and MAPK are two crucial pathways involved in inflammatory responses and in the regulation of the cell cycle. Both pathways can cooperate through shared common receptors and downstream signaling proteins [67,68]. A wide spectrum of compounds contained in postbiotics can directly or indirectly target these pathways, although this does not always result in a similar response. This is a consequence of bacterial strain specificity in the stimulation or attenuation of the inflammatory response, which is a common theme seen in the literature. Nevertheless, the administration of postbiotics seems to be an attractive and promising potential therapeutic intervention to alleviate excessive and/or prolonged inflammatory responses. Many probiotics have shown efficacy in alleviating the symptoms of IBD and other inflammatory gastrointestinal diseases. NF-κB and/or MAPK pathways have been proposed to be the key molecular targets of compounds contained in these probiotics [69,70,71,72].

#### 2.1.1. Molecular Mechanism of NF-κB Signalling

The NF-κB pathway is one of the best-studied intracellular circuits governing inflammatory responses. The transcription factor NF-κB regulates multiple aspects of innate and adaptive immune functions [73]. There are five mammalian members of the NF-κB family of transcription factors: RelA (p65), RelB, c-Rel, NF-κB1 (p50/p105), and NF-κB2 (p52/p100) [74]. NF-κB DNA binding activity consists of many possible homo and heterodimers, although p50/RelA heterodimers are most commonly observed [75]. Under normal cellular conditions, NF-κB binds to and is negatively regulated by an inhibitor of kappa B (IκB) in the cytoplasm. Following an inflammatory stimulus, IκB is phosphorylated by the IκB kinase (IKK) complex and undergoes proteasomal degradation. This allows NF-κB to translocate to the nucleus, where it regulates the transcription of a wide variety of target genes—including chemokines, interleukins, adhesion molecules (i.e., ICAM and VCAM), and antiapoptotic factors such as Bcl-2 and Bcl-xL—and also changes in immune cell activity, for example, the differentiation of T-cells, the maturation of dendritic cells (DCs), or polarization of macrophages. The final outcome of activation may vary depending on the type of activated cell as well as the signal transduction pathway [74]. Importantly, postbiotic elements are recognized directly or indirectly by multiple receptor families; signals originating from these receptors may lead to phosphorylation and subsequent ubiquitin-dependent degradation of IκB.

#### 2.1.2. MAPK Signalling Pathway

MAPKs belong to the large, evolutionarily conserved family of serine/threonine kinases. They consist of three main subfamilies: c-Jun N-terminal kinases (JNKs), extracellular signal-regulated kinases (ERKs), and p38 MAP kinases [76]. The ERK pathway responds mainly to mitogens and growth factors, controlling cell cycle and differentiation [77]. The other two pathways have a wider influence on cellular response. Besides cell cycle and differentiation, JNK and p38 proteins are also engaged in apoptosis, cytokine production, regulation of proliferation, and inflammatory responses [78,79,80].

MAPK pathway signal transduction is based on phosphorylation cascades. The first kinases, MAP kinase kinase kinase (MAPKKK), are activated by a large group of “second-messenger” proteins, such as RAS/RAC proteins, or receptors such as tyrosine kinases (RTKs), G-protein-coupled receptors (GPCRs), integrins, small GTPases Ras and Rap, TNFRs, or pattern recognition receptors (PRRs). MAPKKKs phosphorylate and activate MAPKK kinases (e.g., MKK4 and MKK7), which further phosphorylate MAPK effector kinases (ERK, JNK, and p38). Phosphorylated MAPKs execute cellular responses via multiple protein effectors, such as other kinases or transcription factors [80,81,82].

#### 2.1.3. Postbiotics’ Influence on NF-κB and MAPK Pathways

The effects that postbiotics exert on inflammatory pathways have been studied by numerous groups. Reports have highlighted changes in the transcription of pro-inflammatory genes, modified pro-inflammatory protein expression levels, or changes in overall cell response to stimuli [83,84,85,86]. However, the observed results differ; the reported pro- or anti-inflammatory effects depend on the postbiotic origin, bacterial species or strains [87], or even between cell-free supernatants (CFS) and heat-killed bacteria (HKB) [88]. Both anti-inflammatory and immunostimulatory effects have been demonstrated, suggesting that a heterogeneous structure and composition of different postbiotic media or additional factors influence the final results.

Postbiotics, both from CFS and HKB, mediate anti-inflammatory functions mostly via the attenuation of NF-κB activity [89,90,91,92]. The mechanisms of this attenuation frequently involve the inhibition of NF-κB nuclear translocation via the inhibition of IκB phosphorylation and the prevention of its proteasomal degradation [32,93,94]. Furthermore, the CFS of *L. rhamnosus* CNCM I-4036 was reported to enhance the expression of an IκB subunit, NFKBIA, and thus tighten NF-κB sequestering in the cytoplasm [95].

Both NF-κB and MAPK pathways are triggered by a common group of PRRs, and thus, the downstream events may be affected simultaneously, leading to the attenuated inflammatory response through decreased NF-κB activity and MAPK phosphorylation. This has been observed for HKB and CSF and also isolated postbiotic factors such as 8.7 kDa proteins from *L. plantarum* 10hk2 CFS [96], *L. rhamnosus* LGG-derived DNA [97], HKB of *Weissella cibaria* JW15 [98], and CFS of *L. salivarius* MG4265 [99]. In all of these studies, the decreased NF-κB activity (inhibition of IκBα degradation and/or translocation) and p38 phosphorylation were observed.

The immunostimulatory potential directly connected to NF-κB/MAPK activation has also been reported in the literature. For example, multiple HKB of Lactobacilli strains were able to increase the nuclear translocation of NF-κB subunit p65 [100,101] and to stimulate inflammatory responses. In the case of MAPK, activation by secreted peptides [102,103] and HKB [47,104] has been reported, resulting in the increased production of heat-shock proteins (especially HSP25 i HSP72) and beta-defensin 2 (hBD-2). These may positively influence intestinal barrier integrity and improve the protection of the host against infection [105,106]. Additionally, CFS from *L. reuteri* induced apoptosis via MAPK activation [107].

Finally, some reports show a dual mode of action: the activation of both NF-κB and p38 MAPK signaling. This has been observed with HKB of *L. plantarum* KCTC 13314BP [108], exopolysaccharides (EPS) [109], and S-layer proteins isolated from *L. acidophilus* NCFM [110]. Interestingly, S-layer proteins isolated from *L. acidophilus* NCFM showed different activity depending on the cell type and testing conditions; they stimulated the MAPK pathway in the human adenocarcinoma cell line, HCT116, whereas in the murine macrophage cell line, RAW264, they attenuated NF-κB and MAPK pathway activities [110,111].

**Figure 1:**
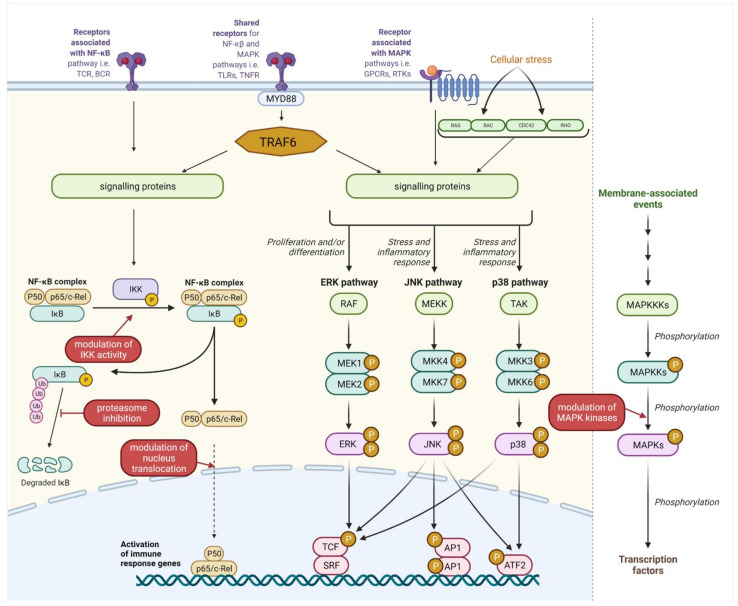
NF-κB and MAPK pathways and their modulation by postbiotic molecules. TCR—T-cell receptors, BCR—B-cell receptors, TLRs—toll-like receptors, TNFR—TNFα receptors superfamily, IKK—IKK kinase complex, GPCRs—G protein coupled receptors, RTKs—receptor tyrosine kinase, MyD88—myeloid differentiation factor 88, TRAF6—TNF receptor associated factor 6, Ras—Ras GTPase, Rac—Rac GTPases, CDC42—Cell division control protein 42, Rho—Rho GTPases. MAPKKKs -mitogen activated kinase kinase kinases which including: RAF, MEKK and TAK kinases, MAPKKs—mitogen activated kinase kinases including: MEK1, MEK2, MKK4, MKK7, MKK3, MKK6. MAPKs—mitogen activated kinases including: ERK—Extracellular signal-regulated kinase, JNK—c-Jun N-terminal kinases, p38—p38 mitogen-activated protein kinases (p38s), AP1—activator protein 1, ATF2—cyclic AMP-dependent transcription factor, TCF—LEF/TCF family of nuclear factors, SRF- serum response factor. Created with BioRender.com, accessed on 10 October 2021.

The vast majority of cited studies have focused on the effects of postbiotics on NF-κB translocation to the nucleus, proteasomal degradation of NFKBIA, or phosphorylation of MAPK kinases (Figure 1). However, the exact influence of postbiotics on downstream signaling pathways needs to be further explored, as their influence is not always obvious and consistent. Moreover, structure–activity assays may provide much better insight into the molecular basis of the described interactions and may help to design more targeted approaches using postbiotic sources.

### 2.2. Other Mechanisms and Molecular Pathways

The intestinal barrier is exposed to multiple harmful factors, which can negatively affect cell survival and disrupt the barrier’s integrity. The influence of probiotic components and metabolites on the regulation of intestinal epithelial barriers has been recently discussed in detail elsewhere [112]. Nevertheless, from a molecular point of view, PI3K/AKT is frequently mentioned as a potential target pathway for postbiotic factors, controlling intestinal barrier functionality. PI3K/AKT modulates cell survival, apoptosis, and intestinal integrity, e.g., mucin production [113]. This pathway may cooperate with NF-κB and MAPK cascades modulating immune responses toward probiotic/postbiotic compounds [114,115,116,117]. The PI3K/AKT pathway is also interconnected with the AMP-activated kinase (AMPK) pathway, a metabolic pathway, which is another cellular effector of probiotics/postbiotics [118,119,120].

The activation of protein kinase B (PKB/Akt) by postbiotics is also prevalent in the literature. PKB plays a role in various cellular responses generally conferring health benefits, such as attenuating inflammatory responses to isolated LGG peptides p40 and p75 [121,122,123], increasing mucin production (or muc2 gene expression) in response to butyrate and purified protein HM0539 from LGG [124,125], increasing the synthesis of tight junction proteins (claudins-3 and 4) in response to butyrate [126] and the CSF of *L. plantarum* RG14 [127].

Interestingly, the modulation of PI3K/AKT by postbiotics was observed in different cell types, which are not present in the intestine environment, thus extending the possible usage of postbiotics as potential drug candidates. For example, the injection of *L. reuteri* extract promoted wound healing in mice; this effect was dependent on PI3K/AKT/β-catenin/TGFβ1 pathway activation [128]. Moreover, the CSF of *L. fermentum* mitigated the induction of the H2O2-induced senescence process in murine adipocytes via the inhibition of the PI3K/Akt/mTOR pathway [129].

## 3. Molecular Targets of Postbiotics

The majority of interactions between bacteria and hosts are mediated by a large heterogeneous group of receptors called PRRs. PRRs recognize molecules frequently found in pathogens, which are called pathogen-associated molecular patterns (PAMPs), or molecules released by damaged cells, called damage-associated molecular patterns (DAMPs) [130]. Documented PRRs that recognize postbiotic molecules include Toll-like receptors [131], nucleotide-binding oligomerization domain-containing protein (NOD)-like receptors [132], and C-type lectin receptors (CLRs) [133]. These receptors do not function in isolation; they cooperate with each other and with various co-receptors [134]. Here, we mainly focus on TRLs and NLRs, whereas the other receptors are only briefly mentioned.

### 3.1. Toll-like Receptors

**Figure 2:**
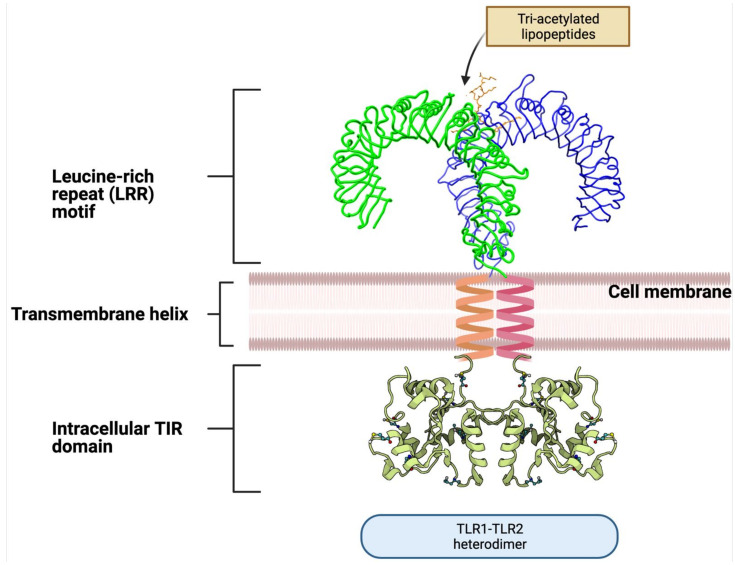
Schematic representation of the TLR structure. The main structural elements include: leucine-rich repeat (LRR) motif; transmembrane helix and intracellular TIR domain. The LRR structure is based on the model of the TLR1-TLR2 heterodimer (PDB ID: 2z7x) interacting with 6 tri-acylated lipopeptides, whereas the TIR domain homology model is based on the TLR2 TIR structure (PDB ID: 1fyw). Based on: [140]. Created with BioRender.com, accessed on 10 October 2021.

Intestinal homeostasis is largely mediated by TLR signaling [135,136] which serves as the main “communication channel” between the host and bacterial species inhabiting the intestine [137]. TLRs are type I membrane receptors present on immune cells (i.e., DCs, lymphocytes, neutrophils, and macrophages), epithelial cells, and neurons. They are localized at the cell surface or intracellular compartments, such as the endoplasmic reticulum (ER), endosome, lysosome, and endolysosome. They play a crucial role in the innate defense mechanisms against invading pathogens [138] and are activated by factors present in postbiotics. TLRs are composed of three structural elements: the cytoplasmic Toll/IL-1 receptor (TIR) domain that initiates downstream signaling [139], a single transmembrane helix, and the N-terminal ligand recognition ectodomain. The ectodomain displays a horseshoe-like structure with leucine-rich repeats (LRRs) that mediate PAMP recognition (Figure 2).

**Figure 3:**
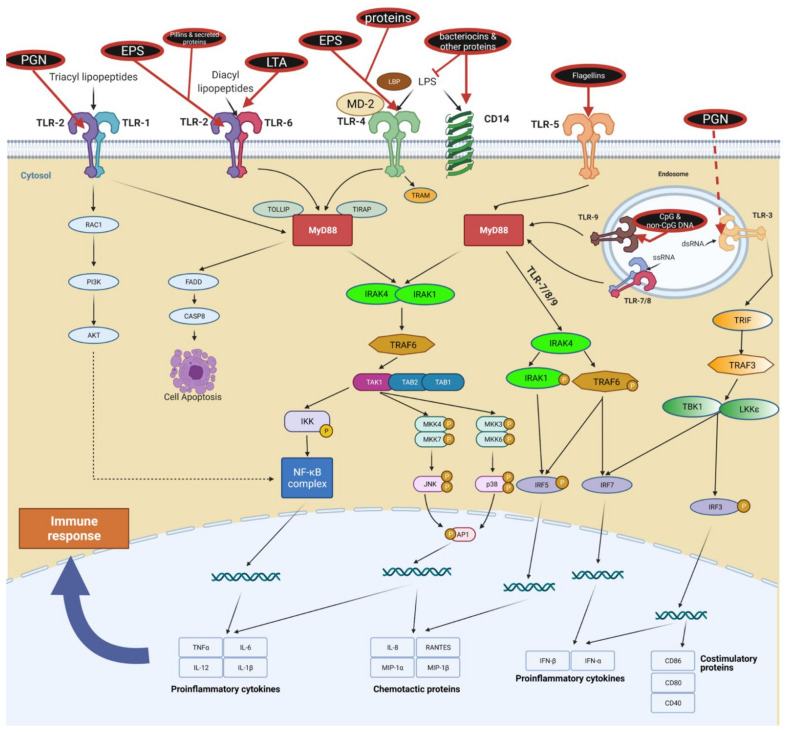
TLR pathways triggered by postbiotic molecules. Postbiotic ligands are presented in black ellipses with red borders and are connected with possible TLR receptors. For bacteriocins, the neutralizing effect on LPS was also highlighted by the red inhibition line. EPS—exopolysaccharides, PGN—peptidoglycan, LTA—lipoteichoic acid, CpG & non-CpG DNA—Unmethylated CpG motifs, LBP—lipopolysaccharide binding protein, TOLLIP—toll-interacting protein, TIRAP—Toll/interleukin-1 receptor domain-containing adapter protein, TRAM—Translocating chain-associated membrane protein, MyD88—myeloid differentiation factor 88, RAC1—Ras-related C3 botulinum toxin substrate 1, PI3K—phosphoinositide-3 kinase, AKT—protein kinase B, FADD—Fas-associated protein with death domain, CASP8—caspase-8, IRAK4/1—Interleukin-1 receptor-associated kinase 4/1, IRF3/5/7—interferon regulatory factor 3/5/7, TRIF—TIR-domain-containing adapter-inducing interferon-β, TRAF3—TNF receptor-associated factor 3, TAK1—TAK1 mitogen-activated protein kinase kinase kinase, TAB1/2—TGF-Beta Activated Kinase 1/2. Created with BioRender.com, accessed on 10 October 2021.

TLRs can exist as monomers (e.g., TLR3, 5, 7, 8, 9, 11) or form homo- and heterodimers (e.g., TLR1, 2, 4, 6); different receptor configurations allow the recognition of diverse PAMPs of bacterial and viral origin. For example, TLR2/TLR1 heterodimers recognize triacyl lipopeptides compared to TLR2/TLR6 that recognize diacyl lipopeptides and LTA [111,112]. They also differ in triggering specific immune responses. TLR2/TLR6 may attenuate inflammatory/regulatory responses, whereas the TLR2/TLR1 complex may exert pro-inflammatory effects [113,114,115]; however, overall immune responses are still under discussion. Both TLR2/6 ligands [116,117] and TLR2/1 ligands [118] can induce opposing immune responses via these heterodimers. The key explanation for the broad diversity of observed results is probably a time-dependent structure–activity relationship between ligands and TLRs. Figure 3 shows the abundance of TLR configurations triggered by postbiotic molecules. Please refer to Table 2 for more details.

TLRs are connected to multiple cellular pathways, such as NF-кB, interferon regulatory factors (IRFs), PI3K/AKT, and MAPK/ERK [142]. The signal is transduced downstream by specific adapter proteins, which have kinase, ubiquitinase, or ligase activity. Both TLR and their adapter proteins (e.g., MyD88) have TIR domains that mediate specific molecular interactions [143,144,145].

Most TLRs transduce signals via the myeloid differentiation factor 88 (MyD88) protein, which is a central node of inflammatory pathways [146]. One exception is TLR3, which mediates signaling directly through the TIR-domain-containing adapter-inducing interferon-β (TRIF) pathway [142]. TLR4 is also unique in that it can activate both MyD88- and TRIF-dependent pathways [142]. This double activation is cooperative and results in the balanced production of both pro-inflammatory cytokines and type I interferons (IFNs), specifically IFNꞵ [142,147,148]. Thus, TLR4 is the only non-nucleic acid-recognizing receptor that triggers a type I interferon response.

Finally, TLRs cooperate with other receptors; for example, TLR activation may be enhanced by the activation of tyrosine kinase Bkt [149] or mediated by endosomal MHC Class II complexes [150,151]. Bacterial peptides are present on MHC class II complexes and may act as adjuvants in TLR-dependent innate responses. Bioinformatics analysis revealed that multiple peptides from commensal bacteria showed an affinity toward MHC complexes, possessing putative immunomodulatory effects [152].

Postbiotic interactions with TLRs seem to be dependent on ligand structure. However, interactions are not restricted to a single ligand–single TLR. Ultimately, the signaling outcome is a result of multiple ligand–receptor events occurring simultaneously, e.g., peptides presented on MHC and LTAs bound to TLR2/TLR6. This overall effect gives rise to a strain-dependent unique immune response. Below we discuss the most prominent ligands, and show how their structural features could be involved in TLR signalling.

#### 3.1.1. TLR Ligands: LTA

One of the most extensively studied ligands present in postbiotics is LTA. Produced by Gram-positive bacteria, LTA is somewhat falsely considered an equivalent of LPS (synthesized by Gram-negative bacteria). LPS mediates the pro-inflammatory response via the TLR4/CD14/MD2 pathway, whereas LTA is mainly recognized by TLR2/TLR6 heterodimers with CD14 and CD36 as coreceptors.

Response to LTA varies from anti-inflammatory to proinflammatory (immunostimulatory) and strongly depends on its chemical and structural composition. For example, LTA from *L. casei* YIT9029 and *L. fermentum* YIT0159 induce a proinflammatory response through TLR2 receptors, which results in TNF-⍺ expression in macrophages [153]. However, LTA from *L. plantarum* exerts an anti-inflammatory action in monocytic cells and decreases TNF-⍺ production [154,155,156].

Multiple structure–activity relationship (SAR) studies have been conducted with LTA derived from different bacterial species (both probiotic and pathogenic) and with synthetic LTAs. Three main structural modifications influencing LTA activity profiles have been revealed [49]. The removal of D-alanine in *L. rhamnosus* GG enhanced the anti-inflammatory potential in murine colitis models [157]. A similar effect was observed by changing D-alanine to glucosyl substitutions in *L. plantarum* [158]. Additionally, decreasing the degree of saturation of acyl chains in the glycolipid moiety of LTA enhanced its anti-inflammatory properties [159]. For structural details, see the summary Figure 6. From the drug discovery perspective, the possibility to select and/or modify LTA to adjust inflammatory effects is encouraging. Although the role of LTAs in modulating immune responses is significant, their influence is too weak in relation to the observed cumulative effect [49].

#### 3.1.2. TLR Ligands: Flagellins

Flagellins are bacterial helical proteins, which are the main structural element of the bacterial locomotion organelle flagellum. They are recognized by TLR5 [160] and by the interleukin-1β (IL-1β) converting enzyme (ICE) protease activating factor (IPAF) [161,162]. The TLR5 receptor is mainly present on the basolateral side of intestinal epithelial cells; thus, by recognizing flagellins, it informs whether bacteria have passed through the intestinal epithelium [163]. TLR5 is strongly engaged in the microbiome–host crosstalk, shaping the overall bacterial community [164] and boosting immune system maturation [165].

TLR5 regulates the production of anti-flagellin immunoglobulins, which help maintain tolerance to commensal bacteria by suppressing flagellin gene expression in many bacterial species. Improper recognition of bacterial flagellins is considered one of the factors associated with IBD [166,167], similar to excessive TLR5 stimulation, which disrupts intestinal barrier integrity [168]. Additionally, a few studies reported that TLR5 may influence metabolic cascades in vivo; TLR5-KO mice, or impaired flagellin recognition, resulted in metabolic syndrome [169], changes in the composition of the intestinal microflora, impaired fat metabolism, and inflammation [170]. However, the exact metabolic influence of TLR5 is still under discussion [171].

Flagellins produced by different strains of lactic acid bacteria seem to have a wide spectrum of immunological activity. For example, flagellins isolated from commensal *L. ruminis* induced pro-inflammatory IL-8 production in human colonic epithelial cell lines via TLR5-dependent mechanism [172]. However, flagellins isolated from a different genus, *L. agilis*, did not enhance IL-8 production in Caco-2 cells. Generally, flagellins isolated from *Lactobacillus* strains were less inflammatory compared to pathogenic strains such as *S. typhimurium*.

These results can be explained by structural differences between the commensal and pathogenic flagellins. Most of the amino acid residues are conserved between flagellins; however, *S. typhimurium* and *L. monocytogenes* have the L-Q-R motif in the recognition site of TLR5, whereas *L. ruminis* and *L. agilis* have the L-G-R and L-N-R motifs, respectively, which decrease the binding and activation of TLR5 [173]. These structural differences are possibly an evolutionary adaptation, present in many commensal bacteria, to decrease immunological activation and reduce clearance by host defense mechanisms [174].

As a postbiotic, flagellins have been shown to mediate pro-health effects. For example, in a state of impaired immunity, flagellins of the probiotic *E. coli* Nissle 1917 increased the expression of the human antimicrobial peptide β-defensin 2 (BD2, also known as DeFB4) by intestinal epithelial cells, enhancing host defense systems against pathogens [175].

Flagellins–TLR5 interactions exert effects on immunological homeostasis through protection against bacterial infection. From this point, the exploitation of TRL5 structural motifs to develop an antagonist or a partial agonist compound could be potentially interesting. A key property of partial agonists is that in the presence of a full agonist (bacterial stimuli), they will compete for the same receptor and thereby reduce the ability to produce the maximum effect.

#### 3.1.3. TLR Ligands: Other Cell Wall-Associated Proteins and Fimbriae

Besides flagellins, other microbial cell-surface proteins may interact with TLRs. Pili (fimbriae) are small, fibrous, surface proteins present in both Gram-negative and Gram-positive bacteria. Mostly characterized in pathogens, they act like virulence factors promoting host–cell adhesion and pathogenesis [176,177]. In commensals, pillins are suggested to provide some beneficial properties to a host [178,179,180,181].

Pili may directly or indirectly interact with TLRs and modulate immune responses. The work on the influence of SpaCBA pili on the immune response in vitro, using different knockout models of *L. rhamnosus* LGG, showed it depends greatly on the interplay between pili and other cell wall elements such as LTA [180].

Furthermore, while the reduction of SpaCBA expression in *L. rhamnosus* LGG elevated IL-8 production in Caco-2 cells, the overexpression of the SpaCBA pilus dampened IL-8 expression [180]. These interactions are mediated via TLR2 receptors. Overall, the authors suggested that pili may function as regulators of IL-8 expression by attenuating the effects induced by other surface molecules, such as LTA.

Finally, an interesting example of an indirect interaction between cell wall-associated proteins and TLRs is the elongation factor Tu (eFTu) and chaperone protein GroeL (also known as GroL). Originating from *L. johnsonii* La1, these two multifunctional proteins were shown to stimulate IL-8 secretion in IECs in a CD14-dependent manner [182,183]. CD14 is a co-receptor that is thought to function as a signal amplifier by inducing TLR4 endocytosis and activating TRIF-dependent pathways, leading to the production of type-I IFNs [184,185].

#### 3.1.4. TLR Ligands: Oligonucleotides

Unmethylated CpG motifs found in bacterial and viral DNA, double-strand RNA (dsRNA), single-strand (ssRNA), and specific oligonucleotide sequences are present in high amounts in the intestine [186]. These PAMPs act as activators of the endosomal TLR9, present in multiple immune cells (e.g., DCs and B cells), in IECs, and also in various lung cells [187,188,189]. TLR9 is especially engaged in shifting immunological responses between Th1 (INF-γ producing) and Th2 (mainly producing IL-4) lymphocytes and in the maintenance of immunological homeostasis in the intestine [136].

The immunomodulatory effect of unmethylated CpG motifs depends on their structure, origin, and physiological conditions. Some of the most notable features of CpG motifs influencing TLR9 activation are thymidine content, flanking sequences, backbone type, presence of a 5′-TC, poly-G sequence, length, and concentration [190]. These are used to design synthetic oligonucleotides with targeted activity, such as adjuvants in vaccines [191,192].

To date, a few probiotic CpG motifs have been characterized, mostly from *Lactobacilli* and *Bifidobacterium*. *L. rhamnosus* LGG-derived oligonucleotides display immunostimulatory potential by increasing the IFN-γ/IL-4 ratio. This skewed the lymphocyte differentiation from Th2 to Th1 and improved intestinal barrier function in a mice model of allergy [193]. This is in line with previous experiments, where LGG CpG motifs induced the Th1 response and the production of cytokines (INF-γ, IL-6, IL-12, IL-18, and TNF-α) [194,195]. The GTCGTT sequence, which has been proposed to have strong immunostimulatory effects, is frequently present in the genomes of *L. casei*, *L. plantarum*, *L. rhamnosus*, and *Bifidobacteria* [196]. It has been proposed that genomic DNA contributes to about 50% of the immunomodulatory effects of living probiotics [197,198]. Some studies using isolated CpG motifs provide insight into the mechanisms of modulating allergic responses of probiotic strains [199,200]; however, human studies with CpG motifs as single ligands are still lacking.

However, probiotic-derived oligonucleotides may also exert an anti-inflammatory effect, for example, via the inhibition of NF-кB in intestinal epithelial cell lines [97]. Another report showed that gDNA and CpG motifs significantly decreased the LPS-induced IL-6 mRNA levels in RAW264.7 macrophages [201]. Importantly, oligonucleotides derived from bacteria can differ in activity due to the experimental conditions, such as the presence or absence of inflammatory stimuli. Namely, the pretreatment of THP-1 monocytic cells with *L. plantarum* gDNA attenuated the pro-inflammatory effect of LPS administration. However, when administered alone, gDNA had the opposite effect [202].

In summary, similar to other postbiotic TLR ligands, oligonucleotides have a wide spectrum of activity. Depending on the origin, sequence/structure, and physiological conditions, they may augment or attenuate inflammatory responses.

#### 3.1.5. TLR Ligands: Bacteriocins

Antimicrobial peptides (AMPs), or bacteriocins, are one of the secreted protein fractions produced by bacteria. AMPs are secreted to fight other bacteria competing for the same ecological niche [203]. AMPs can also act as signaling (quorum sensing) or colonizing peptides, and importantly, they can shape the overall microbiome community [204]. The ability to synthesize AMPs is a desirable feature of probiotic strains, which influences their survival in the human gastrointestinal tract [204,205,206].

In higher organisms, AMPs are important components of innate immunity, protecting the host against infections. The presence of commensal AMP-producing strains in intestines was confirmed by biochemical [207,208] and genetic studies [209,210]. Notably, AMP-producing strains seem to be predominant in the human microbiome [207,211].

Multiple studies have reported the immunomodulatory potential of AMPs, which enhance or attenuate immune responses in the gut at multiple levels of the cell’s signaling pathways [212,213,214,215,216,217,218]. Most frequently, the observed outcome was a result of an indirect effect of AMPs on TRLs, that is, through binding (and neutralizing) their natural ligands (i.e., lPS, LTA, lipoproteins, and oligonucleotides) [219]. Bacteriocins, which are mostly cationic, bind to anionic elements of the bacterial cell wall (i.e., LPS) through electrostatic interactions [141]. For example, an increase in hydrophobic properties via amino acid substitution enhanced the binding of human cathelicidin CAP18/LL-37 analogues to LPS [220]. Nisin, a bacterial AMP, may bind and neutralize LPS [221]. This effect may explain previously observed immunomodulatory results obtained with Nisin and other bacteriocins tested in the presence of LPS [222,223,224,225,226,227]. Similarly, human AMP, LL-37, neutralizes LPS, reducing the activation of the TLR4 and TLR2/1 heterodimers [214,228]. Interestingly, LL-37 is also engaged in direct interactions with cell surface receptor CD14 [220,229,230], suggesting the involvement of both direct and indirect mechanisms of action. Recently, local administration of nisin in an animal model of clinical endometritis lead to alterations in the cytokines’ (B7-2, IFN-γ,IL-2, and IL-8) expression profile and prevented *Staphylococcus aureus* infection-induced endometrial changes in uterine tissue, similarly to administration of kanamycin [231].

Taken together, the administration of bacteriocins may be a promising alternative or complement to classic antibiotics. These properties, such as LPS-neutralizing potential and anti-microbial activity, seem to be strictly connected to structural features.

#### 3.1.6. TLR—Not Only Intestine Receptors

The role of TLRs in health and disease is not restricted to immunological reactions. TLR4 and TLR2 were found on intestinal nervous cells and smooth muscle cells, influencing intestinal motility [232,233]. TLR4 and TLR5 are proposed as two main receptors engaged in liver inflammation [234], diet-induced liver diseases [235], and hepatocellular carcinoma [236,237,238]. Postbiotic ligands such as bacteriocins may cross the intestinal barrier and act beyond the intestines [239,240].

TLRs are one of the most engaged receptors in sensing postbiotic-derived factors. Bacterial proteins, peptides, LTA derivatives, polysaccharides, and nucleic acids can all trigger TLR signaling. The exerted physiological effect is a result of the diverse interactions that a “cocktail of molecules” provides and may have a negative or beneficial influence on human health. The key factor determining postbiotic ligand activity seems to be strain specificity. In addition, TLRs are not only intestinal or immunological receptors; they also play physiological roles in other tissues. These properties open new routes for the possible application of postbiotic molecules as potential drug candidates.

### 3.2. NOD like Receptors (NLR)

NOD receptors (nucleotide-binding oligomerization domain-like receptors, nucleotide-binding leucine-rich repeat receptors, or NLRs) are crucial intracellular receptors involved in innate immune responses. NLRs are capable of interacting with various PAMPs present in postbiotics [241,242,243]. NLRs’ interaction with the host microbiome was recently extensively reviewed [244]. Here, we focus on the two main postbiotic sensors, NOD1 and NOD2, and briefly introduce inflammasomes as important communication hubs between the host and microbiome.

**Figure 4:**
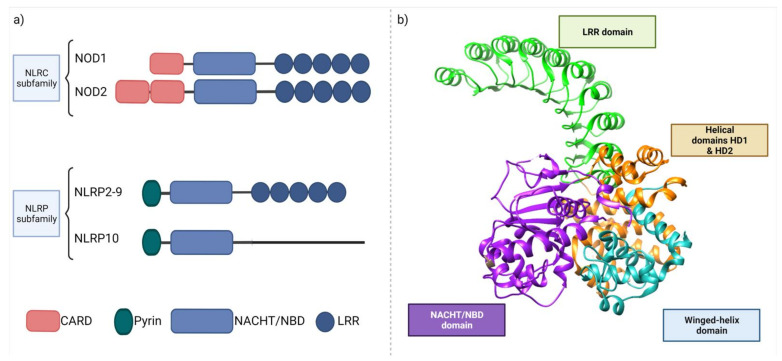
Structure of the NLR receptors. (**a**) Structural differences between the NLR protein family. NOD1/2 are members of the NLRC subfamily containing the CARD domain. The pyrin domain is characteristic for the NLRP family, whose main members are inflammasomes, and can additionally differ in the LRR region. (**b**) NOD2 receptor structure (PDB ID: 5iam) with ribbon representation of the main domains: LRR (leucine rich repeats), NACHT/NBD (nucleotide binding domain) and Helical domains. Created with BioRender.com, accessed on 10 October 2021.

The human NLR family consists of 22 proteins, including NOD-like receptors and several proteins serving as scaffolds in forming multiprotein complexes termed “inflammasomes” [245,246]. NLRs are expressed in many cell types, including immune cells (mainly macrophages and neutrophils) and epithelial cells. Their expression can differ between cells, e.g., NOD1 is expressed in multiple cell types comprising the intestinal epithelium and DCs, whereas NOD2 is not detected in intestinal epithelial cells [247]. NOD1 and NOD2 (Figure 4) are both multi-domain proteins consisting of a variable N-terminal effector region, caspase recruitment domain (CARD), a centrally located NOD domain that is critical for activation (known as NACHT or NBD), and C-terminal LRRs that sense PAMPs [132]. The main structural difference between NOD1 and NOD2 is the number of CARD domains, being single in NOD1 and double in NOD2 [248].

**Figure 5:**
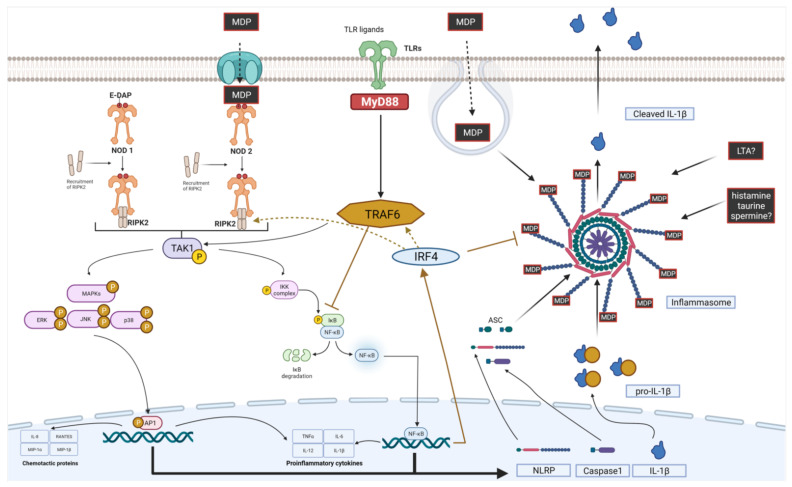
The interaction between TLR and NLR cascades in recognition of postbiotic ligands (black squares with red border). The signalling cascade of NOD1/2 receptors with TLR and inflammasome pathways is shown. NOD2 signaling cascade increases expression of IRF4 (yellow arrow), which binds to TRAF6, inhibits poly-ubiquitination of RIPK2 kinase (yellow dash arrows) and inhibits NF-κB activity (yellow inhibition line). IRF4 may also have suppressor activity against inflammasomes, described in [250]. NOD2 receptors are able to regulate the expression of inflammasomes constituents: NLRP, Caspase1 and IL-1β [251]. Created with BioRender.com, accessed on 10 October 2021.

Contrary to TLRs, NOD-like receptors are located intracellularly and recognize ligands, which translocate into the cytosol after phagocytosis and degradation in phagolysosomes. NOD1 recognizes γ-d-glutamyl-meso diaminopimelic acid (DAP) from Gram-negative bacteria, whereas NOD2 can detect muramyl dipeptide (MDP) from both Gram-positive and Gram-negative bacteria [249]. NOD1 and NOD2 are activated through direct ligand binding, which leads to self-oligomerization. This allows for the interaction of the N-terminal domain with receptor-interacting serine/threonine protein kinase 2 (RIPK2), which in turn induces pro-inflammatory response cascades via MAPK and NF-κB pathways (Figure 5).

#### 3.2.1. NOD and the Microbiome Crosstalk

NOD receptors are crucial PRRs in communication between the host and microbiome. NOD1 receptors are involved in recognizing and shaping the bacterial community by regulating the host immune response through AMP synthesis [252]. Knocking out Nod1 leads to the disruption of intestinal barrier integrity, enhanced inflammatory response, and inflammation-mediated tumorigenesis. Notably, the depletion of gut microbiota suppressed the observed pathology [253]. NOD2 regulates the number, size, and T-cell composition of Peyer’s patches (PPs), which are a part of gut-associated lymphoid tissue (GALT) [254]. In turn, T-cells present in the PP can modulate paracellular and transcellular permeability, and as a result, bacteria migrate from the gastrointestinal tract to extraintestinal sites [254,255]. NOD2-deficient mice showed an altered microbiota composition [256,257], increased transcellular permeability, and pathological changes in immune cells [255,258]. Notably, mutations in Nod2 genes are associated with increased susceptibility to Crohn’s disease (CD) [259,260], highlighting its immune–microbiome crosstalk association.

#### 3.2.2. Other Regulatory Roles of NOD Receptors

Both NOD1 and NOD2 seem to be implicated in other processes beyond immune responses. From a therapeutic point of view, modulation of insulin homeostasis seems promising, where NOD2 may play a protective role, contrary to NOD1. For example, knocking out Nod1 lowered the levels of macrophage-induced adipose tissue inflammation and neutrophil recruitment [261,262]. However, depletion of NOD2 receptors had the opposite effect; it enhanced adipose tissue and liver inflammation and exacerbated insulin resistance [263]. Similar conclusions were drawn from studies where the NOD1 ligand (FK565) induced whole-body insulin resistance, whereas the NOD2 ligand (peptidoglycan) had a protective effect on mice fed a high-fat diet [261,263]. This effect depends on interferon regulatory factor 4 (IRF4) activation, which plays a role in NOD2, but not in the NOD1 signaling cascade (Figure 5) [264]. The activation of NOD2 leads to increased IRF4 expression and binding to TNF receptor-associated factor 6 (TRAF6) and RIPK2. This inhibits the polyubiquitination of TRAF6 and RIPK2 and results in the down-regulation of NF-κB [265]. Recently, the selective activation of RIPK2 has been proposed as the second molecular mechanism of NOD2-mediated improvement in glucose tolerance. These effects were observed on non-hematopoietic cells and suggest that this mechanism is cell-type dependent [266].

#### 3.2.3. NOD and Postbiotic Molecules

**Figure 6:**
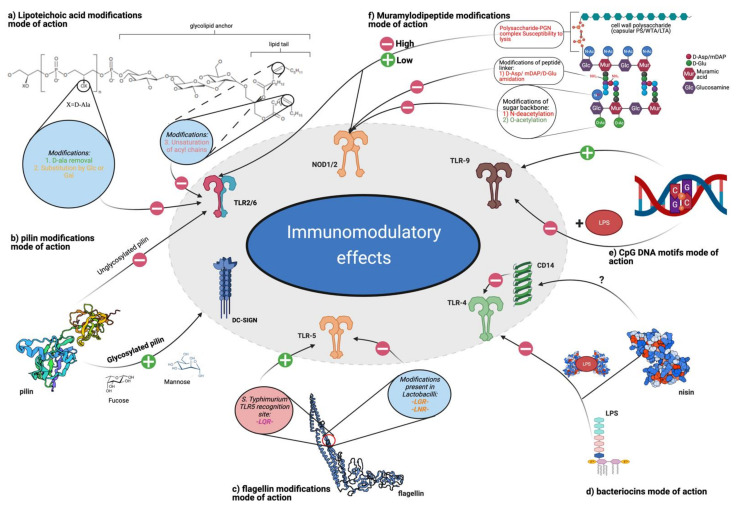
Postbiotic ligands mode of action and their modification, which affects their activity. Plus or minus symbols on arrows symbolize a possible influence on the target receptor; plus = activation/enhanced activation; minus = reduced activation/blockade. (**a**) Lipoteichoic acid modification: presented structure based on LGG’s LTA from [134]. Described modifications mainly influence TLR2 signalling. (**b**) Pillin modifications: pilin structure based on SpaA pili from LGG (PDB ID: 5f44). (**c**) Flagellin modifications mode of action: structure based on flagellin from *S. thypimuirum* (PDB ID: 1ucu) modifications presented in commensal *Lactobacilli*, reduced activation of the TLR5 receptor. (**d**) Bacteriocin mode of action: nisin (PDB ID: 5xhb) is presented as an example of bacteriocin with LPS-binding potential, which reduces activation of TLR4. Interaction between CD14 and LL-37 has been reported, lack of similar data for bacterial AMPs. (**e**) CpG DNA motifs mode of action: presence of inflammatory agent may change CpG activity from immunostimulatory to anti-inflammatory. (**f**) Muramyldipeptide modifications mode of action: structure based on review of cell wall structure in LAB strains [335]. Presented modification of sugar backbone lowers lysozyme degradation and NOD1/2 activation. Resistance to lysis of intact cell wall (polysaccharide-peptidoglycan complex) defines immunomodulatory potential. Created with BioRender.com, accessed on 10 October 2021.

Several studies suggest that the bacterial cell wall component, peptidoglycan (PGN), and NOD receptor interactions are greatly responsible for the strain-specific effects of probiotics [267,268,269]. After the administration of PGN into immunocompromised mice, the respiratory and systemic adaptive humoral responses measured as cytokine levels improved [270,271]. Systemic stimulation of NOD1 by microbiota-derived PGN led to enhanced systemic immunity [252]. A growing body of evidence suggests that the observed immunological effects are directly connected with the chemical structure of PGN. Below, we summarize some of the modifications in the structure of PGN defining the immunomodulatory potential of bacteria (Figure 6).

PGNs have a polymeric structure and contain layers of muramyl dipeptide (MDP). The obligatory unit of MDP recognized by NOD2 is N-acetylmuramic acid (MurNAc) [272]. Multiple bacteria can modify the structure of MDP, which helps to reduce PRR presentation and immune system activation. The known modifications include the following:

- N-deacetylation of the sugar backbone; this is the most common modification in Gram-positive bacteria [273], which reduces lysozyme degradation and the activation of NOD and the NLRP3 inflammasome [273,274].
- The amidation of the ε-carboxyl group of meso-diaminopimelic acid (mDAP) or the amidation of the α-carboxyl group of glutamic acid reduces lysozyme degradation and NOD1 activation. This has been reported for some *L. plantarum* strains [275,276].
- O-acetylation of MurNAc reduces the degradation of PGN by lysozymes, resulting in the lower presentation of MDP fragments and reduced NOD signaling [273,277].
- The polysaccharide-PGN complex (connected by phosphodiester bond), described for the first time in L. casei Shirota (LcS) [278], suppresses IL-6 production via the inhibition of NF- κB phosphorylation and enhances NOD2 mRNA levels in RAW264 macrophages in vitro [279]. Furthermore, it provides resistance to lysis, which determines amplified IL-12 production in macrophages. This effect is observed only for intact cell walls consisting of PGN and cell wall polysaccharides. Purified PGN has a weak ability to induce IL-12 production, whereas minimal structural units of PGN (6-O-stearoyl-muramyl dipeptide) abolish the effect of intact cell walls [247].

The above-mentioned influence of polysaccharide-PGN on the production of IL-12 was shown to be mediated by both TLR2 and NOD2, suggesting a dual mode of action of PGN [280]. However, direct (and selective) interaction between PGN and TLRs is still debated [281]. *L. salivarius* Ls33 induces the development of CD103+ DCs and CD4+ Foxp3+ regulatory T cells in an IL-10-dependent manner without any TLR involvement [50]. However, PGNs from *L. rhamnosus* CRL1505 exert their immunostimulatory potential via TLR3 [282]. TLR2 and NOD2 interplay in PGN recognition, which is crucial in response to many Lactobacilli strains [268,283]. Despite this controversy, the above studies indicate that the structure of PGN defines recognition and affinity to its biological target(s).

#### 3.2.4. Inflammasomes and Postbiotic Molecules

Besides NOD receptors, the other group classified as NLR proteins are inflammasomes. Inflammasomes are multiprotein, cytosolic innate immune signaling complexes that sense different stimuli and may lead to the activation of caspase 1. They are expressed in multiple cell types and are able to recognize both intrinsic and extrinsic stimuli. The best-described inflammasomes involved in microbiome–host interplay are NLRP3, NLRP6, and NLRP12 [284].

NLRP3 seems to play a protective role in IBD [285], colorectal cancer [286], and maintaining proper balance in the gut environment via the regulation of IL-1β and AMP secretion [287]. NLRP6, preferentially expressed in goblet cells and enterocytes, is involved in similar processes: host defense against infection, autoimmune responses, tumorigenesis, and intestinal homeostasis. However, how and when NLRP6 modulates host–microbiome crosstalk is still under discussion [283].

Inflammasomes are also engaged in systemic processes, i.e., renal inflammation (NLRP3 and NLRP6) [288,289] and the gut–brain axis (NLRP3) [290]. It was shown that NLRP12 may have a systemic influence on microbiome composition, leading to various metabolic imbalances. Knocking out the NLRP12 gene causes a significant reduction of the SCFA-producing *Lachnospiraceae* phylum, which may provoke obesity and the development of metabolic syndrome [291].

Inflammasomes are sensitive to microbiome ligands. NLRP3 seems to be engaged in butyrate sensing as a hub protein mediating negative symptoms in IBD [292]. NLRP6 recognizes LTA from Gram-positive bacteria and shares common ligands with TLRs. The activation of NLRP6 by LTA, originating from pathogenic bacteria, leads to excessive IL-18 release, which exacerbates infection [293]. This is caused by the suppression of NF-κB signaling, which in turn attenuates the inflammatory response, i.e., dampens cytokine, chemokine, and cell surface protein expression. Interestingly, the NLRP6–LTA interaction leads to an excessive inflammatory response, but mild stimulation may be protective [294].

Microbiota-associated metabolites, such as taurine, histamine, and spermine, may modulate NLRP6 inflammasome signaling, epithelial IL-18 secretion, and downstream AMP profiles, resulting in the improvement in barrier integrity [295]. However, similar to the LTA case, histamine dosage is crucial; excessive exposition may induce an inflammatory response mediated via the histamine 2 receptor [296,297]. In summary, inflammasomes are engaged in recognizing postbiotic ligands and are an important part of overall response to PAMPs. They can recognize small molecules (e.g., amino acids) and more complex structures like LTA or LPS. More research is needed to elucidate a more clear structure–activity relationship between ligands and NLRPs [298].

#### 3.2.5. NLR and TLR Interplay

There is a high degree of bidirectional crosstalk between NLR and TLR receptors, which fine-tunes the final immune response (Figure 5). NODs and TLRs can act in tandem by recognizing different bacterial ligands and transmitting signals through intestinal cells. They activate similar hub proteins responsible for the inflammatory response (NF-кB, AP-1, and IRFs), which results in an augmented and more targeted response to bacterial infection. NOD ligands, such as DAP and MDP, along with ligands recognized by TLRs, exert a synergistic effect on the expression of proinflammatory cytokines in human immune cells [256,299,300,301]. 

However, in vivo studies suggest a more complicated interplay. In mice overexpressing NOD2 receptors, the administration of MDP reversed increased transcellular permeability induced by TLR2 (agonist: Pam3CSK4) and TLR4 (agonist: LPS). In NOD2-KO mice, TLR2 and TLR4 expression was elevated in basal conditions, which suggests the involvement of NOD2 in the regulation of TLR expression [255]. These results indicate that NOD2 may also dampen the inflammatory activity of TLR pathways, which results in a more balanced overall immune response.

Both TLRs and NLRs are also engaged in intestinal angiogenesis, and bacterial ligands for NOD1/2 and TLR2/4/6 stimulate the production of angiogenic factors. Knocking out RIPK2 and TRAF6 (a common hub protein for both pathways) inhibited the observed results [302]. 

NLR and TLR cellular effects are rather analogous, as both families act through similar and well-known pathways. The picture changes, however, when in vivo studies are conducted. Where cellular and tissue interplay occurs, a less obvious, dose-dependent, bifurcated, and bidirectional interplay is unraveled (Figure 5). 

### 3.3. Other Receptors

There are a number of receptors expressed on intestinal cells that may interact with bacterial molecules (Table 3). SCFRA receptors (GPR43, GPR41, GPR109a, and Olfr78), the pregnane X receptor (PXR), and the aryl receptors (AhR) are all interesting targets from the drug discovery point of view. However, due to the new definition of postbiotics, where metabolites are no longer classified under this term [23], they are only briefly described in Table 3. In this section, we introduce c-type lectin receptors (CLR) with a special focus on DC-specific ICAM3-grabbing non-integrin (DC-SIGN) receptors, which is the best-described member of this family, regarding postbiotic recognition.

#### C-Type Lectin Receptor (CLR)—DC-SIGN

CLR are PRRs that recognize specific motifs from bacteria, viruses, and fungi. The family consists of 17 different subgroups classified according to structural differences with a common domain: lectin. The lectin domain can recognize a wide spectrum of glycans from pathogens, commensals, or host proteins. Under some circumstances, CLRs can also recognize certain proteins and lipids [313]. CLRs are expressed mainly on myeloid cells and to a lesser extent on other immune cells [314,315]. They play a role in immunological processes, such as cell–cell adhesion, autophagy, and apoptosis. CLRs also have the ability to orchestrate immune responses to commensal and pathogenic bacteria by regulating gene expression and modulating TLR signaling [133,313,316,317,318]. They are one of the biggest groups of PRRs, engaged in recognition of a whole spectrum of pathogen and commensal associated molecules. They are also less studied when compared to NLRs or TLRs.

The most commonly described receptor, considering postbiotic recognition, is DC-SIGN. This transmembrane receptor is present on intestinal cells (especially myeloid- and monocyte-derived DCs), endothelial cells, and specific subpopulations of macrophages [319,320,321]. It recognizes high mannose- and fucose-containing regions on polysaccharides and proteins, which leads to the activation of serine/threonine kinases RAF-1, the acetylation of the induced NF-κB subunit p65, and finally affects cytokine expression, e.g., the upregulation of IL-10 [322].

According to the above work, bacterial exopolysaccharides (EPS) rich in mannose and fucose seem to be natural ligands for DC-SIGN. Unfortunately, it is difficult to draw clear conclusions concerning DC-SIGN–EPS interactions. This is due to different isolation and purification methods. There are also no studies confirming the direct involvement of DC-SIGN receptors in the mentioned interaction [323,324]. Nevertheless, in many studies on EPS [325,326,327,328], an increase in the production of IL-10 has been observed, which may indicate involvement of CD-SIGN/RAF-1/p65. Studies on EPS revealed that the main receptors engaged in EPS-immunomodulatory properties are TLR2 or TLR4 [329]. Similar observations have been published for *Bifidobacterium animalis* subsp. lactis [324] and for LGG-derived EPS [57]. At this moment, these findings do not rule out the engagement of DC-SIGN or DC-SIGN-TLR tandem [330].

Importantly, glycosylation may affect the activity of various postbiotic ligands. For example, in the case of pili, which are TLR ligands, glycosylation expands their affinity toward DC-SIGN receptors [331]. Indeed, mannose and fucose residues on SpaCBA pili from *L. rhamnosus* GG can interact with DC-SIGN receptors on DCs [331]. This leads to the induction of different types of cytokines compared to non-glycosylated pili recognized by TLR2 (Figure 6).

S-layer proteins have been reported as other DC-SIGN ligands [46,47]. S-layers form two-dimensional protein arrays that are located at the outermost surface of bacteria walls [332]. They exert multiple biological functions, including cell wall synthesis, control of cell division, mechanical and osmotic stabilization, pH balance, and bacteriophage protection [332,333]. They possess high structural diversity and are extensively glycosylated. The structure of S-layer proteins defines their affinity to DC-SIGN receptors or TLR/DC-SIGN tandems [45,334]. Moreover, downstream cytokine expression differs between different types of S-layer proteins; *L. acidophilus* NCFM SlpA induces IL-10 and IL-12p70 production, whereas SlpB induces IL-12p70, TNF, and IL-1 [334].

## 4. Conclusions

Postbiotic ligands constitute a big heterogenous group of compounds with different physico-chemical and structural properties. These properties are a result of a long evolutionary adaptation and can be unique to the phylum and/or strain of bacteria. The underlying mechanisms of observed responses are governed both by the structural properties of ligands and their concentration.

Postbiotic ligands interact with host cells via a plethora of routes. These include extracellular (e.g., TLRs, C-lectin) and intracellular (NLRs) pattern recognition receptors. PRRs act in cooperation, adjusting the cellular response to recognized ligands and creating a diverse, strain specific outcome. Furthermore, the effects of particular postbiotic molecules may be dependent on the experimental background, testing conditions and/or disease model. The combination of these factors could create a specific “context of action” by modulating receptor gene expression, ligand sensitivity and immune response, that finally results in a diverse outcome of postbiotic–host interactions.

However our mechanistic knowledge about the whole “cocktail” of molecules present in HKB/CFS postbiotic fractions is still far from complete. Currently there are a few already commercially available pharmaceutical preparations based on postbiotics: CFS-like products Colibiogen^®^ (Laves-Arzneimittel GmbH, Schötz, Switzerland), Hylak^®^ Forte (Ratiopharm/Merckle GmbH, Germany) and cell-wall lysate—CytoFlora^®^ (BioRay Inc., Laguna Hills, CA, USA). All are multicomponent and multi strain derived products without a defined exact composition and/or molecular mechanism of action. Further studies on postbiotic–host interactions should focus on defining types of active molecules present in HKB/CFS, their structure–activity relationship (SAR), affinity to multiple PRRs, and finally, their effect on host cells driven by the interplay of PRRs.

Postbiotics not only interact with host cells, they may also exert effects on microbial communities, which is an indirect mechanism of action. Bacteria influence each other’s existence through nutrient sharing and/or scavenging and cell-to-cell communications, functioning organizationally like tissues from higher organisms [336,337]. Disruption of the ecological organization of the normal gut microbiota (dysbiosis) can be associated with numerous chronic human disorders. Postbiotics promise to modulate these communities with specific molecules (e.g., bacteriocins, quorum sensing peptides, small organic molecules), influencing the community composition and/or its metabolic profile [338]. Overall, postbiotics are a promising alternative to probiotics/synbiotics and antibiotics, especially for GI tract infections, where restoring microbiome homeostasis is crucial.

From a drug technology point of view, the key advantage of postbiotics lies in their reduced risk of bacteremia [28]. In addition, compared to classical probiotics, postbiotics do not have issues with product shelf survival, and do not require special conditions of storage. Recent findings show that genetic modifications of specific bacterial strains can result in molecules capable of specific functional effects, as seen with single modification of LTA, which dampens or enhances inflammatory reactions.

In summary, postbiotics are a promising source of active molecules with an evolutionary optimized mechanism targeting not only immunological or metabolic systems, but also the microbiome community.

## Figures and Tables

**Table 1:** Postbiotic elements present in supernatants or in heat-killed/inactivated bacteria.

Postbiotics and Biologically Active Bacterial Byproducts	
Supernatant-Derived (CFS)	Cell-Derived (HKB)
- Peptides: - bacteriocins [29] - other peptides (quorum sensing, cyclic) - proteins: - proteases i.e., lactocepin [30,31]. - cell wall hydrolases i.e., p40 & p75 molecule [32] - serine protease inhibitors (i.e., serpin) [33] - other extracellular proteins [34,35] - lipids: - SCFA (i.e., butyrate [36] - conjugated linoleic acid (CLA) [37] - other less studied fatty acid derivatives - other exopolysaccharides - small organic molecules - lactic acid - D-amino acids [38] - indole derivatives [39,40] - other unknown small molecules - vitamins/cofactors [27] - inorganic molecules i.e., polyphosphate [41,42]	- S-layer proteins [42,43,44,45,46,47,48] - lipoteichoic acid (LTA)—as derivative of cell wall component [49] - peptidoglycan-derived muropeptides from cell wall [50] - polysaccharides from cell wall - galactose-rich polysaccharides [capsular PS] [51,52,53,54,55,56,57,58,59] - intracellular proteins
- fermentation products * [43]	

* For fermentation products see [58,59].

**Table 2:** The PRRs, subcellular localization, and recognized ligands. Adapted from [141].

Receptor	Receptor Localization	Ligand	Origin of Ligand
TLR2	Cell surface	Lipoteichoic acid	G (+) bacteria
		Lipoprotein/lipopeptides	Various pathogens
		Hemagglutinin protein	Viruses (Measles Virus)
		Glycosyl-phosphatidylinositols	Parasites (Toxoplasma gondii)
		Exopolysaccharides	G (+) bacteria
TLR2/1	Cell surface	Triacyl lipopeptides	Bacteria and mycobacteria
TLR2/6	Cell surface	Diacyl lipopeptides	Mycobacteria
		Zymosan	Fungi
TLR3	Cellular compartment (endosomes)	dsRNA	Viruses
TLR4	Cell surface	Lipopolysaccharide	G (−) bacteria
		Envelope proteins	Viruses (Respiratory Syncytial Virus)
		Glycosyl-phosphatidylinositols	Parasites (Toxoplasma gondii)
TLR5	Cell surface	Flagellin	Bacteria
TLR7/8	Cellular compartment (endosomes)	ssRNA	Viruses
TLR9	Cellular compartment (endosomes)/cell surface	CpG-containing DNA	Bacteria and viruses
TLR11	Cell surface	Uropathogenic bacteria component	Bacteria (uropathogenic Escherichia coli)
		Profilin	Parasites
NOD1	Cell cytoplasm	Meso-diaminopimelic acid	PGN of G (−) and some G (+) bacteria
NOD2	Cell cytoplasm	Muramyl dipeptide (MDP)	PGN of G (+) and G (−) bacteria

G (+), Gram-positive; G (−), Gram-negative; dsRNA, double-stranded RNA; ssRNA, single-stranded RNA; PGN, peptidoglycan; Meso-DAP, γ-D-glutamyl-meso-diaminopimelic acid; MDP, muramyl dipeptide NOD1, nucleotide oligomerization domain-like receptor 1; NOD2 nucleotide oligomerization domain-like receptor 2.

**Table 3:** Other possible host receptors and their postbiotic ligands.

Receptor	Ligand	Outcome	Reference
EGFR	p40 and p75 plantaricin P1053	phosphorylation of Akt and ERK kinases; increased viability of non-cancerogenic cells	[303,304,305]
formyl peptide receptors (FPRs)	N-formyl-Met-Leu-Phe; HK Lactobacillus	ERK phosphorylation without stimulating pro-inflammatory phospho-IκB or pro-apoptotic phospho-c-Jun NH2-terminal kinase	[306]
formyl peptide receptor-like 1 (FPRL1)	human bacteriocin LL-37	upregulated angiogenesis via increased intracellular calcium levels	[307]
CCR6	human bacteriocins beta-defensins	increased chemotaxis of dendritic and T cells	[308]
pregnane X receptor (PXR)	Indole derivatives (i.e., Indole-3-propionic acid)	improved tight junction functions by increased expression of claudins and occludins	[39]
aryl hydrogen receptors (AhRs)	indole-3-lactic	increased Il-22 expression	[40]
Short chain fatty acid receptors (GPR43, GPR41, GPR109a and Olfr78)	Short chain fatty acids (i.e., butyrate, propionate)	modulates AMP-kinase and NF-кkB activity regulates glucose homeostasis, immune processes, permeability of cell membrane, oxidative status, cell cycle or brain functions	[309,310,311,312]
